# Phytoplankton morpho-functional trait dataset from French water-bodies

**DOI:** 10.1038/s41597-021-00814-0

**Published:** 2021-02-02

**Authors:** Christophe Laplace-Treyture, Jonathan Derot, Emilie Prévost, Anne Le Mat, Aurélien Jamoneau

**Affiliations:** 1 INRAE, UR EABX, 50 avenue de Verdun, F-33612 Cestas, Cedex France; 2 Pôle R&D Ecosystèmes Lacustres (ECLA), F‐13100 Aix‐en‐Provence, France; 3 Estuary Research Center, Shimane University, 1060 Nishikawatsu-cho, Matsue, Shimane 690-8504 Japan

**Keywords:** Limnology, Freshwater ecology, Community ecology

## Abstract

In lake ecosystems, phytoplankton communities can be studied by adopting taxonomic-based approaches. However, these approaches suffer from identification issues and are sometimes of limited ecosystem ecological value. The recent development of functional approaches may allow an evaluation of other aspects of ecosystem quality, functions and interactions with abiotic parameters or other communities. Here, our aim was to create a phytoplankton trait database at the French scale. This database will be relevant for the analysis of phytoplankton communities that will lead to a better understanding of phytoplankton functional ecology in lakes of France and other European countries possessing similar biological communities. We used a French national database of phytoplankton occurrences sampled from 384 lakes over the entire French metropolitan territory. A final list of 636 taxa was used to compile 53 morpho-functional traits associated with taxonomic information. The traits encompassed variables such as shape, biovolume, motility, toxin production and Reynolds groups. With this new database, we aim to provide data for morpho-functional analyses of phytoplankton assemblages from local to European scale.

## Abstract (summary)

**Table:** 

Measurement(s)	morphological feature • functional • biovolume • life form • geometric cell form • cell size • carbon content • size • cell motility • mucilage • presence of heterocyte • plastid • presence of siliceous skeleton • cyanotoxin • protuberance • chlorophyll b • chlorophyll c • xanthophyll • nutrition mode • reproduction • trophic water class • Tolerance • Reynolds group • presence of lorica • presence of plate • akinete formation • number of flagella
Technology Type(s)	digital curation
Sample Characteristic - Organism	freshwater phytoplankton species
Sample Characteristic - Environment	lake • freshwater lake
Sample Characteristic - Location	French River Lakes

Machine-accessible metadata file describing the reported data: 10.6084/m9.figshare.13416659

## Background & Summary

In water bodies, phytoplankton communities are key elements of ecosystems, regulating biogeochemical cycles^1^ and representing one of the most important photoautotrophic primary producer^2,3^. Because of their rapid responses to water quality and anthropogenic pressures, phytoplankton communities are commonly used as trophic and ecological indicators^4,5^ in Europe and in the United States^6^. Therefore, they are mainly studied using a classical taxonomic-based approach, i.e. abundance and biovolume of species. Taxonomic lists are used as a whole, with the sum of individual biovolume employed for total biomass metric assessment^7,8^, and at the species level for selecting indicator species. This selection is usually based upon the most common species of the phytoplankton community, for which information and ecological profiles can be established^9^. Therefore, the assessment is only based on a part of the community. Rare species are not considered in such cases although they can be very informative^10^ (e.g. new invasive species with very specific response to a trophic or a polluted state). Data can be aggregated at higher taxonomic level (family or phylum) to take into account all available taxa^7,11^. However, different taxa of the same phylum can indicate a very different ecological status while species from different phylum can reflect very similar ecological conditions^3^. Moreover, the ecological significance can be neutralized at the phylum level by species indicative of opposite conditions^12^.

During the last decades, approaches based on functional groups were created to overcome these drawbacks^13–15^ and allow evaluating other aspects of ecosystem quality, functioning and biotic interactions^16,17^. As outlined in Salmaso *et al*.^18^ many studies use functional classifications and/or functional groups with the objective to better investigate links between phytoplankton communities and the main environmental factors. Some classifications mentioned by Salmaso *et al*.^18^ are based on one characteristic (size classes) or several (morphologically based classifications) while others use functional properties (functional or morpho-functional groups). Indices based on functional groups were also published for lakes^19^ and for rivers^20^ to be used for the implementation of Water Framework Directive, WFD^21^. Nevertheless, as explained by Padisák *et al*.^22^ functional groups were not built using all encountered taxa. Ecologists have to adjust their groups manually or through an R code for the Reynolds Functional Groups^23^. These additions, may not be adequate and lead to misinterpretations. According to Wentzky *et al*.^24^, the use of functional-traits at the species level (and not at higher taxonomical level) can limit these issues without influencing any assumption about taxon functional group.

Thus, data collections of phytoplankton traits at the species level are required for a large number of taxa. Accordingly, different datasets were created considering the taxa size. Olenina *et al*.^25^ reported size-classes (PEG biovolume, http://ices.dk/data/vocabularies/Documents/Forms/AllItems.aspx) but mainly for marine waters. Kremer *et al*.^26^ established a dataset for freshwater and estuaries for a large number of taxa (>1200 species), which had the advantage that it considered cell size and natural unit size (colonial and filamentous growth forms). At the French level, Druart and Rimet created a trait dataset including only biovolumes^27^ and published, more recently, a phytoplankton trait dataset^28^ mainly based on Alpine lake surveys and including around 10 compiled traits.

To our knowledge, specific traits such as lorica, protuberance presence (and kind of protuberance), potential toxicity associated with potential toxin production by cyanobacteria, heterocyte and vacuole presence have never been merged together in one trait database. More generally, to our knowledge, no database covers a large number of ecological and morphological traits for phytoplankton communities at the French or European level. To be able to use many morpho-functional traits for water bodies in ecological analyses in France, the construction of such a dataset was initiated. This was based upon the survey database established for the WFD application on French water bodies since 2005. This dataset will be particularly helpful for studying the relationships between functional traits and environmental variables, as well as to develop new trait-based indicators for water quality assessment.

We aim in this paper to (1) compile morpho-functional traits of phytoplankton taxa in French water bodies (metropolitan territory) (2) offer information of the corresponding phylogeny (3) provide a dataset helpful for analyzing functional and morpho-functional phytoplankton assemblages from local to national scale (4) deliver a dataset allowing consistent data comparisons between European countries.

## Methods

### Site description

**Fig. 1:**
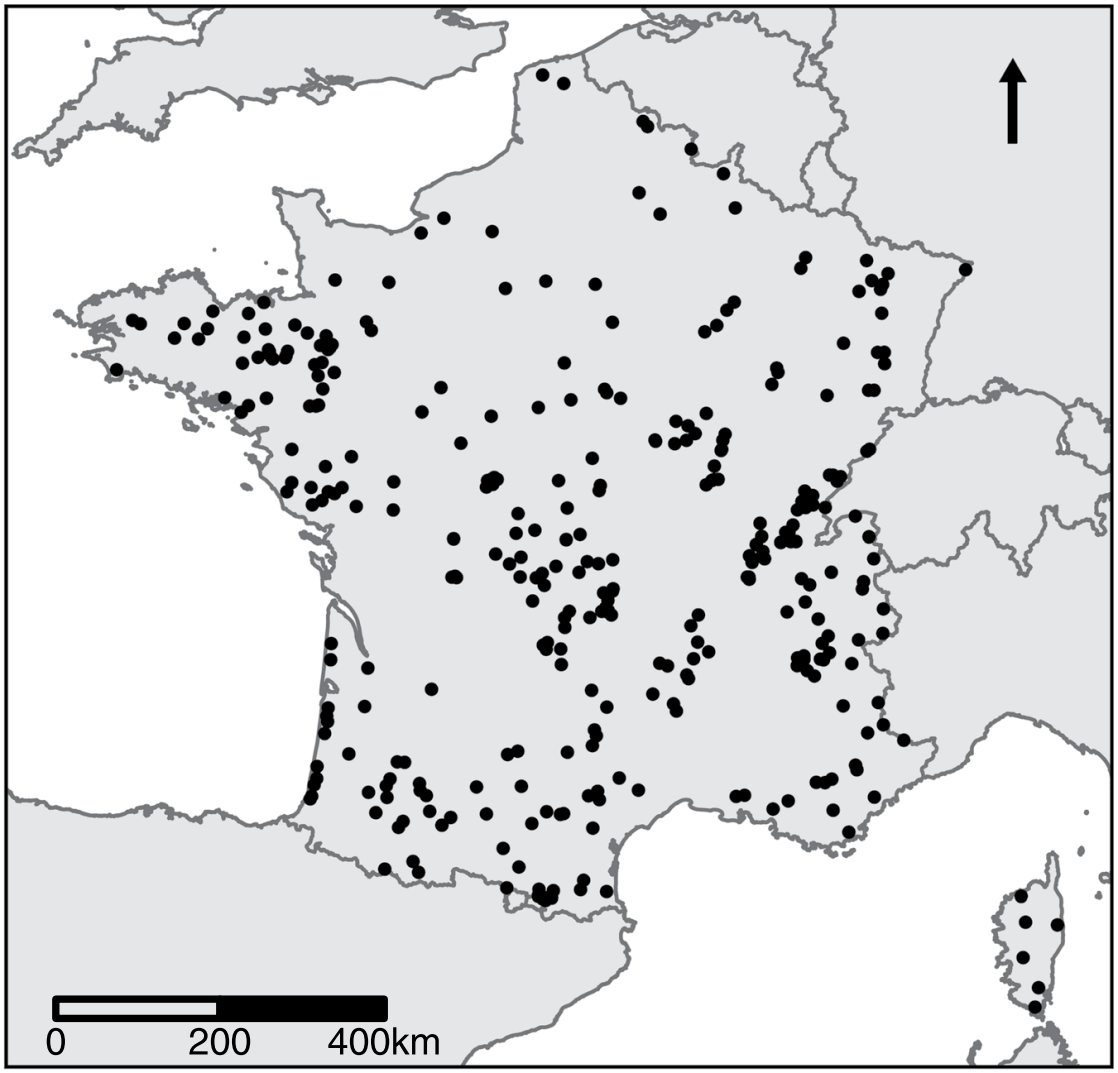
Location of the 384 lakes covered by our study; each black dot represents a lake sampled between 2005 and 2016.

This work was based on the French national database consisting of phytoplankton communities surveyed in 384 lakes between 2005 and 2016 and used for applying the Water Framework Directive. As each lake has been sampled several times this database contains 2,987 phytoplankton samples. Sampled lakes cover the whole French territory (Fig. 1) and encompass all natural and artificial types of lakes encountered in France: from lowlands to alpine location, from small to large lakes (0.09 to 577.12 km^2^), from shallow to deep lakes (mean depth from 0.30 to 153 meters) and also from oligotrophic to hypereutrophic environmental conditions.

### Data acquisition and compilation

In order to create our dataset we firstly defined the taxa list recorded in the national database. The extracted list was firstly examined for taxonomic consistency. Taxa only determined at a high taxonomic level (order, class, phylum) were rejected from the list. Thus, genus and below this level (species, variety, form, etc.) were conserved to obtain an accurate taxonomical list for morpho-functional traits (e.g. biovolume, cell form). A taxa name in the national database can be different over time because of taxonomic changes and determination by different operators. To harmonize taxa denomination and to obtain one up-to-date taxa list, the original names used in the lake database were corrected following the most recent research reported on the AlgaeBase website (https://www.algaebase.org/ - last consultation on 31^st^ July 2020) and in scientific papers. This will allow the traits table to be used for future studies. Thus, a list of 1,318 taxa, associated with abundance and biovolume data, was obtained from the database. In order to remove idiosyncratic species and any potential determination errors, we only kept taxa which occurred in more than 15 samples. A final taxa list of 636 taxa was obtained.

Secondly, we selected a list of 53 morpho-functional traits that we considered to be the most relevant for the description/analyses of functional phytoplankton communities (see Online-only Table 1 for detail of these traits). We compiled available trait data from published sources: we used general books (John *et al*.^29^; Reynolds^3^; Wehr *et al*.^30^) for all taxa and specific references listed in Table 1 for more precise information of some traits or taxa. The traits not available in the literature (biovolume, size class, carbon contents, etc.) were determined/calculated. All available synonyms of the taxa recorded in the literature were taken into account in order to consider morpho-functional traits taken from as a many taxonomic references as possible. Taxonomic information was essentially obtained from the AlgaeBase website.

**Table 1:** Specific books and references used for some traits and taxa for the different phyla.

Phylum	References
chlorophytes	Huber Pestalozzi and Thienemann^52^; Komárek *et al*.^53^ and for desmids group: Coesel and Meesters^54,55^
chrysophytes	Starmach^56^
cyanobacteria	Komarek and Anagnostidis^57,58^; Komárek^59^ and for the toxicity: Whitton ed.^39^; Dittman *et al*.^40^; Sanseverino *et al*.^41,42^; Meriluoto *et al*.^43^; Anses^60^
diatoms	Bey and Ector^61^; Cox^62^; Druart and Straub^63^; Houk^64^; Houk and Klee^65^; Houk *et al*.^66–68^; Krammer and Lange-Bertalot^69–72^; Lange-Bertalot *et al*.^73^; Siver *et al*.^74^
dinophytes	Popovsky and Pfiester^75^; Moestrup and Calado^76^
euglenophytes	Huber-Pestalozzi^77^
xanthophytes	Ettl^78^; Rieth^79^

From these publications we mainly obtain morphological characteristics such as cell form, presence of ornamentation on cell and number of chloroplasts. If the information was divergent between two references, the most recent is accepted.

To facilitate the use of the dataset in statistical analyses, taxa code (6 letters in upper case), used in the free computer software Phytobs^31^ (commonly used in France for freshwater phytoplankton counting), was added for each taxon. These codes were constructed with the first 3 letters for genus and the last 3 for species and infra specific denomination. In order to have a global clustering of phytoplankton taxa (sometimes useful for result presentations), more or less independent of phylogeny updates and thus stable in time, we included a phytoplankton group name. We classified phytoplankton taxa in 9 groups based on common clustering of algae and cyanobacteria such as chlorophytes (green-algae), diatoms, xanthophytes (brown-algae), chrysophytes (gold-algae).

The trait life form was added to the dataset and is defined as the common living form of the taxon in its natural environment (cell, colony or filament). Because some taxa could exhibit a second life form in nature, this information was also indicated in the dataset.

**Fig. 2:**
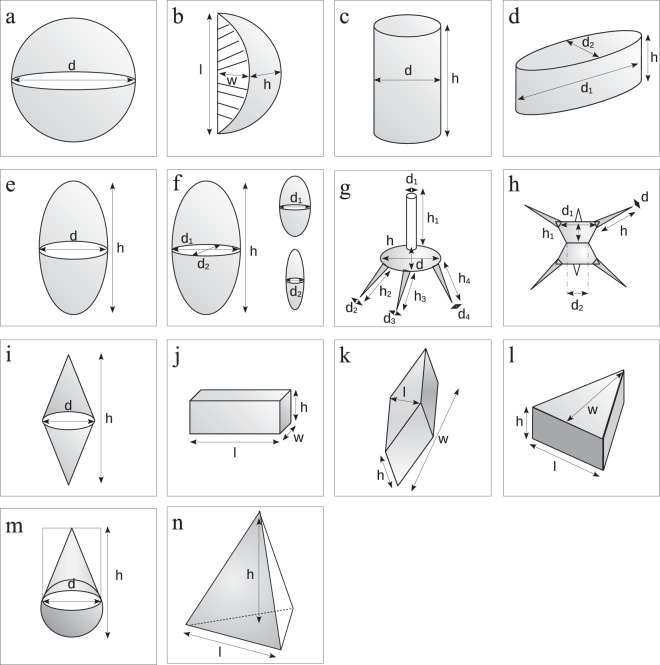
Illustration from Phytobs^31^ of the 14 cell and individual forms used for the biovolume calculations. (**a**)sphere, (**b**) cymbelloid, (**c**) cylinder, (**d**) oval cylinder, (**e**) rotational ellipsoid, (**f**) flattened ellipsoid, (**g**) ceratium and (**h**) staurastrum form, (**i**) double cone, (**j**) parallelepiped, (k) prism on parallelogram base and on (**l**) triangulare base, (**m**) cone with half sphere and (**n**) tetrahedron.

We determined the cell and individual form traits for each taxon with the use of 14 simple geometric forms: sphere, cylinder, rotational ellipsoid, flattened ellipsoid, cymbelloid, staurastrum and ceratium form, oval cylinder, double cone, parallelepiped, prism on parallelogram base and on triangulare base, cone with half sphere and tetrahedron as defined in the computer tool Phytobs^31^ and illustrated in Fig. 2. Choices were made with the help of book taxa illustrations and according to Hillebrand *et al*.^32^, Olenina *et al*.^25^, Druart and Rimet^27^, Hutorowicz^33^ and Padisak and Adrian^34^.

The minimum and maximum cell dimensions were taken from the literature and were used for the definition of the size class (see below).

We determined taxa biovolume following the European standard NF EN 16695^35^ with the use of the software Phytobs^31^ for the calculation (this counting software provides also a biovolume calculation tool). Thus, from real samples observed during the period of the study, 30 individuals of each taxon were measured, as recommended by the European standard, to calculate a mean cell biovolume. For taxa not observed in enough quantity, common minimum and maximum dimensions were taken from the literature in order to determine mean dimensions and calculate a corresponding mean cell biovolume.

We also specified the mean carbon content per cell in our dataset. We used the formulas described in Menden-Deuer and Lessard^36^ and reproduced in European standard NF EN 16695^35^ because they consider the decrease in specific carbon content with cell size. Thus, the general formula used for phytoplankton is Carbon [pg C] = 0.216 × Biovolume^0.939^ and the specific formula for diatom taxa is Carbon [pg C] = 0.288 × Biovolume^0.811^.

Three size class systems were also subsequently defined in the dataset. The first two use the Sieburth *et al*.^37^ class limits and are based on the cell dimensions. The third one uses the biovolume thresholds defined by Ignatiades^38^. For Sieburth *et al*. class limits, two variables were defined: one using the minimal individual length and the second using the maximal individual length. In each case, taxa were classified within the following classes: picophytoplankton (0.2–2 µm), nanophytoplankton (2–20 µm), microphytoplankton (20–200 µm), mesophytoplankton (0.2 mm-20 mm). The Ignatiades classification was calculated on individual biovolume: cell biovolume for unicellular taxa and individual biovolume for colonial and filamentous taxa. The classes defined by Ignatiades were: nanoplankton (10–1,000 µm^3^), microplankton (1,000–1,000,000 µm^3^), macroplankton (>1,000,000 µm^3^) plus a specific class, picoplankton (<10 µm^3^), used for the very small taxa.

Different binary traits (1-presence; 0-absence) were added: motility, presence of flagella, aerotopes, contractile vacuoles, mucilage, akinetes, heterocytes, cysts, plasts, sheath, tractus, siliceous skeleton, lorica, external plates, scales, ornamentations, protuberances, chlorophyll-b and c, xanthophyll, phycobilin and potential toxin production^39–43^ (with toxin family specified: microcystin, anatoxin-a, anatoxin-a(S), cylindrospermopsin, saxitoxin, Beta-Methylamino-L-Alanin). These characteristics were mainly extracted from the literature. The precise number of plastids and flagella were also added. Some distinctions were defined for the protuberance type, their size and their number onto the individuals. The protuberances were split into seven different types including granule, needle or bristle and the size was divided in two size classes (large and small).

The main nutrition mode was indicated for each taxon following the common definitions: autotroph, mixotroph and heterotroph^44,45^.

The reproduction mode was specified more precisely when mentioned in the literature or just indicated as sexual or asexual when not.

Some taxa are known only from freshwater and others also from the marine environment. Thus, this information was indicated as a Water_Type in the trait dataset. The trophic status of the water where the taxon lives was defined under “Water_Trophy” as from oligotrophy to hypereutrophy. In the case of a taxon with a large trophic range, the main trophic status was indicated.

In some literature and mainly in Reynolds^3^, the tolerance to abiotic parameters are defined. We also indicated this trait as a tolerance trait in the dataset (literal form).

We finally reported in the dataset the Reynolds functional groups, based on the original description of Reynolds^3^ and developed further (with more taxa) by in Borics *et al*.^20^ and Padisak *et al*.^19,22^.

## Data Records

The phytoplankton morpho-functional traits dataset^46^ is in the form of a large table with taxa in rows and their corresponding morpho-functional traits in columns. This trait table is contained in a formatted file with semicolon-separated values (*.csv) and stored in a public repository available on https://data.inrae.fr/dataverse/eabx. with the following DOI: https://doi.org/10.15454/GJGIAH. The name of the file was defined as FRENCH_PHYTOPLANKTON_TRAITS.csv. This trait table is designed to be updated with new data especially from French lakes (new surveys and new taxa occurrences) and/or new morpho-functional traits relevant for the study of phytoplankton communities.

In the table some variables are numerical and continuous such as biovolumes or cell dimensions, other are categorical such as life forms and other boolean as toxin production. Consequently, the table is a mixed format with numeric and text. The different variables are listed with their attributes in Online-only Table 1 to facilitate understanding and reuse of the data. Missing information was labelled ‘#NA’.

**Fig. 3:**
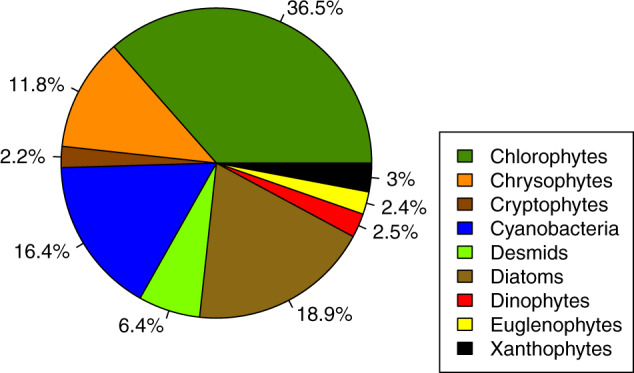
Proportion of the dataset taxa in the different phytoplankton groups.

**Fig. 4:**
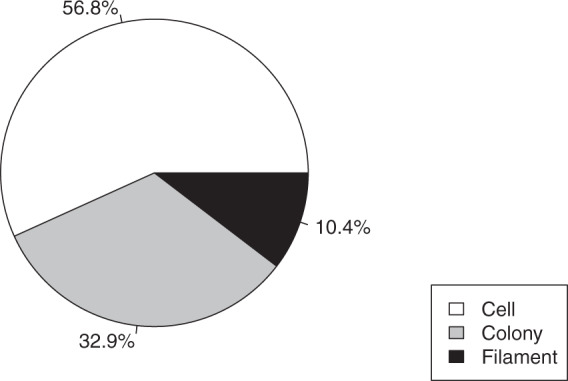
Proportion of the dataset in the different life forms. These represent the usual individual living form of the taxa (cell, colony or filament).

In the dataset, the chlorophytes group is the most represented with 36.5% of the total (Fig. 3) and cryptophytes is the least (2.2%). Other groups like cyanobacteria, diatoms and chrysophytes represent between 10 to 20% of the dataset taxa. The individual life form of each taxon listed is mainly represented by the cell form (56.8%), followed by the colonial form (32.9%) and, finally, the filamentous form of around 10% (Fig. 4).

## Technical Validation

**Fig. 5:**
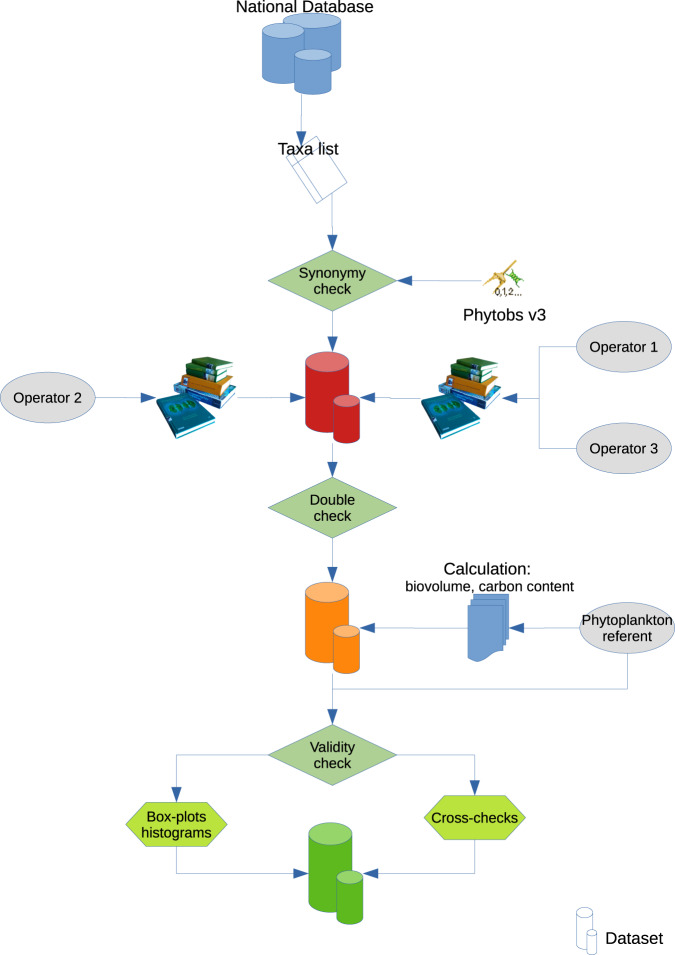
Scheme for the technical validation of the dataset. Extraction of the taxa list from the national phytoplankton database, simplification with the synonymy stored in Phytobs software. Implementation of the traits by 3 different operators to obtain a beta version of the dataset, then double-check step and implementation of the calculated traits to obtain a first version of the dataset. Quality control of the data by the referent scientist through the use of graphs and cross-checks to obtain the final version of the dataset.

The technical validation of the data stored in the dataset was undertaken following the scheme outlined in Fig. 5. All the information collected from the literature was double-checked and all sources are provided in the methods section above. All measures (biovolume, carbon content, size classes attribution, etc.) were made by the same individual in order to reduce error.

After that, all data included in the traits table were controlled by the same person (C.L.T.), an expert on French phytoplankton. The dataset thus created was explored through preliminary analyses with box-plots and histograms in order to check distribution and to highlight erroneous data (outliers, aberrant text, missing data, etc.). Many cross-checks were undertaken in order to highlight missing and inconsistent data (C.L.T.):

- motility set to “1” when aerotope, contractile vacuole or flagellum are present;

- number of flagella indicated with presence of flagellum;

- absence of flagellum for desmids, diatoms and cyanobacteria;

- compulsory flagellum for cryptophytes;

- number of plastids indicated with the presence of plastids;

- absence of plastids for cyanobacteria taxa;

- presence of akinete and heterocyte only for cyanobacteria taxa;

- compulsory cyst for chrysophytes and dinophytes taxa;

- siliceous skeleton only for diatoms;

- presence of external plates only for dinophytes;

- presence of lorica for chrysophytes and few chlorophytes and euglenophytes;

- protuberance type other than “None” and protuberance size not null with presence of protuberance;

- presence of chlorophyll-b and xanthophyll and also absence of chlorophyll-c and phycobilin for chlorophytes (equivalent cross-controls for the other phytoplankton groups were done);

- presence of toxin only for some cyanobacteria;

- the toxin family indicated with presence of toxin.

## Usage Notes

The phytoplankton trait dataset is freely available and stored in https://data.inrae.fr/dataverse/eabx public repository and should be appropriately referenced by citing the present paper. A survey taxa list should be first checked for synonymy before being analyzed with the morpho-functional dataset. This will avoid the mismatch of taxa names. We strongly encourage users to use the synonymy database present in the Phytobs software.

Note that many traits were obtained from literature indicating general characteristics of species or genera and do not represent individual responses to environmental conditions. Some of them can be affected by different environmental conditions. For example, traits based on dimensions (min, max length and width), biovolume, carbon content and in some cases size-classes can be directly influenced by environmental conditions^36,47–49^ as well as the presence of heterocyte and akinete for some cyanobacteria taxa^50^.

## Data Availability

The software Phytobs, used for some trait compilation or calculation (biovolumes), was created by our team in a first version in 2009 by Hadoux and Laplace-Treyture^51^ for phytoplankton counting purposes. It was upgraded over the years to allow biovolume calculations and to integrate taxonomy and synonymy^31^. This free software is now in version 3.2 (French, English and Spanish languages) and publicly available on https://hydrobio-dce.inrae.fr/phytobs-software/.

## Online-only Table

**Online-only Table 1:** List of trait variables and their corresponding attributes (blank if not pertinent) available in our dataset. * When the data are not available, cells contain ‘#NA’.

Variable identity	Variable definition	Unit	Storage type	Range (numeric value) or value (text value)*	Precision (for numeric)
Taxa_Name	Latin name of the taxa		String	(n = 636)	
Author	Author(s) of the name		String	(n = 636)	
Phytobs_Taxa_Code	6 letters code specific to the taxa from Phytobs^31^		Character (6)	(n = 636)	
Phytoplankton_Group	General group of the taxa based on common and global clustering of phytoplankton taxa		String	Chlorophytes Chrysophytes Cryptophytes Cyanobacteria Desmids Diatoms Dinophytes Euglenophytes Xanthophytes (n = 9)**	
Phylum	Phylum of the taxa from AlgaeBase website (https://www.algaebase.org/)		String	Bacillariophyta Bigyra Charophyta Chlorophyta Choanozoa Cryptophyta Cyanobacteria Euglenozoa Haptophyta Miozoa Ochrophyta #NA (n = 12)	
Class	Class of the taxa from AlgaeBase website (https://www.algaebase.org/)		String	Bacillariophyceae Bikosea… (n = 24)	
Order	Order of the taxa from AlgaeBase website (https://www.algaebase.org/)		String	Aulacoseirales Bacillariales… (n = 58)	
Family	Family of the taxa from AlgaeBase website (https://www.algaebase.org/)		String	Achnanthaceae Achnanthidiaceae… (n = 109)	
Genus	Genus of the taxa from AlgaeBase website (https://www.algaebase.org/)		String	(n = 242)	
Species	Species of the taxa from AlgaeBase website (https://www.algaebase.org/)		String	(n = 478)	
Life_Form	Common life form (cell, colony or filament)		Character(4)	Cel. Col. Fil. (n = 3)	
Life_Form2	Secondary life form (sometimes used by the taxa)		Character(4)	Cel. Col. Fil. None (n = 4)	
Phytobs_Cell_Form	Simple geometric form of the cell determined by us. Also reported in Phytobs^31^		String	Ceratium form Cone with half sphere Cylinder Cymbelloid Double cone Flattened ellipsoid Oval cylinder Parallelepiped Prims on parallelogram base Prism on triangulare base Rotational ellipsoid Sphere Staurastrum form Tetrahedron #NA (n = 15)	
Phytobs_Ind_Form	Simple geometric form of the individual determined by us. Also reported in Phytobs^31^. (if individual is cell type then this form equals Phytobs_Cel_Form)		String	Ceratium form Cone with half sphere Cymbelloid Cylinder Double cone Flattened ellipsoid Oval cylinder Parallelepiped Prims on parallelogram base Prism on triangulare base Rotational ellipsoid Sphere Staurastrum form Tetrahedron #NA (n = 15)	
Min_Width	Minimal width of the cell of the taxa	µm	Float	0.4, 128 (n = 636)	0.1
Max_Width	Maximal width of the cell of the taxa	µm	Float	0.7, 169 (n = 636)	0.1
Min_Lenght	Minimal length of the cell of the taxa	µm	Float	0.4, 350 (n = 636)	0.1
Max_Lenght	Maximal width of the cell of the taxa	µm	Float	0.8, 1,000 (n = 636)	0.1
Cell_Biovolume	Cell biovolume calculated by Phytobs^31^	µm^3^	Float	0.3, 571,016 (n = 636)	0.1
Ind_Biovolume	Individual biovolume calculated by Phytobs^31^. If Life_Form equals “Cell” then Ind_Biovolume equals Cell_Biovolume	µm^3^	Float	0.3, 571,016 (n = 503)	0.1
Carbon	Carbon content per cell based on formulas from Menden-Deuer et Lessard^36^	pg	Float	0.07, 18,379.93 (n = 636)	0.01
Min_Size_Ind	Minimal size class of the taxa (individual) according to Sieburth *et al*.^37^		String	Picophytoplankton (0.2–2 µm) Nanophytoplankton (2–20 µm) Microphytoplankton (20–200 µm) Mesophytoplankton (0.2mm-20mm) (n = 4)	
Max_Size_Ind	Maximal size class of the taxa (individual) according to Sieburth *et al*.^37^		String	Picophytoplankton (0.2–2 µm) Nanophytoplankton (2–20 µm) Microphytoplankton (20–200 µm) Mesophytoplankton (0.2mm-20mm) (n = 4)	
Ignatiades_Size_Class	Size class of the taxa based on individual biovolume according to Ignatiades^38^ with the add of one class for the very small one below 10µm3		String	Picoplankton ( < 10µm3) Nanoplankton (10–1,000µm3) Microplankton (1,000–1,000,000µm3) Macroplankton ( > 1,000,000µm3) (n = 4)	
Motility	Capacity of the taxa to be motile		Boolean	0 1 (yes)	
Flagellum	Presence of flagella		Boolean	0 1 (yes)	
Nb_Flagellum	Number of flagella		Integer	0, 1, 2, 4 (n = 4)	1
Aerotope	Presence of aerotope		Boolean	0 1 (yes)	
Contractile_Vacuole	Presence of contractile vacuole		Boolean	0 1 (yes)	
Mucilage	Presence of mucilage around cell(s)		Boolean	0 1 (yes)	
Akinete	Presence of akinete		Boolean	0 1 (yes)	
Heterocyte	Presence of heterocyte		Boolean	0 1 (yes)	
Cyst	Possibility to produce cyst		Boolean	0 1 (yes)	
Plast	Presence of plast(s) in the cell		Boolean	0 1 (yes)	
Nb_Plast	Number of plast in the cell		String	0, 1, > 1, 2, > 2, 8 (n = 6)	
Sheath	Presence of sheath around cells		Boolean	0 1 (yes)	
Tractus	Presence of tractus in the colony		Boolean	0 1 (yes)	
Siliceous_Skeleton	Presence of siliceous skeleton		Boolean	0 1 (yes)	
Lorica	Presence of lorica around the cell		Boolean	0 1 (yes)	
Plate	Presence of plate around the cell		Boolean	0 1 (yes)	
Scale	Presence of scale around the cell		Boolean	0 1 (yes)	
Ornamentation	Presence of an ornamentation on the cell		Boolean	0 1 (yes)	
Protuberance	Presence of protuberance on the cell		Boolean	0 1 (yes)	
Protuberance_Type	Type de protuberance		String	Bristle Cone Granule Needle Ribs Tooth Tractus None (n = 8)	
Protuberance_Size	Size classes of the protuberance		String	Large Small None (n = 3)	
Nb_Protuberance	Number of protuberance		String	0, 1, 2, > 2, 3, > 3, 4, > 4, 5, 6, > 6, 7, 8, 15, 16, 18, 25 (n = 17)	
Chlo_B	Presence of chlorophyll-b as secondary pigment		Boolean	0 1 (yes)	
Chlo_C	Presence of chlorophyll-c as secondary pigment		Boolean	0 1 (yes)	
Xanthophyll	Presence of xanthophyll as secondary pigment		Boolean	0 1 (yes)	
Phycobilin	Presence of phycobilin as secondary pigment		Boolean	0 1 (yes)	
Toxin	Possibility to produce toxin(s)		Boolean	0 1 (yes)	
Microcystin	Potential producer of microcystin		Boolean	0 1 (yes)	
Anatoxin-a	Potential producer of anatoxin-a		Boolean	0 1 (yes)	
Anatoxin-a(S)	Potential producer of anatoxin-a(S)		Boolean	0 1 (yes)	
Cylindrospermopsin	Potential producer of cylindrospermopsin		Boolean	0 1 (yes)	
Saxitoxin	Potential producer of saxitoxin		Boolean	0 1 (yes)	
BMAA	Potential producer of Beta-Methylamino-L-alanin		Boolean	0 1 (yes)	
Nutrition	Mode of nutrition (organic matter or inorganic nutrient)		String	Autotrophic Heterotrophic Mixotrophic (n = 3)	
Reproduction	Main reproduction type of the taxon		String	Asexual Asexual-binary fission Asexual-binary fission or akinete Asexual-fragmentation Sexual Sexual-anisogamy Sexual-cytogamy Sexual-isogamy Sexual-oogamy Sexual and asexual (n = 10)	
Water_Type	Living environment		String	Freshwater Freshwater-marine water (n = 2)	
Water_Trophy	Main trophic water class associated with the taxon		String	Oligotrophic Oligo-mesotrophic Mesotrophic Meso-eutrophic Eutrophic Hypereutrophic (n = 6)	
Tolerance	Resistance of the taxon to a specific range of a parameter		String	Calcium richness Carbon deficiency, light Current speed… (n = 27)	
Reynolds_Group	Reynolds groups according to Reynolds^3^ and actualized by Borics *et al*.^20^, Padisak *et al*.^19,22^		Character(3)	A, B, C, D, E, F, G, H1, J, K, Lm, Lo, M, N, P, Q, R, S1, T, Tb, Tc, U, W1, W2, Ws, X1, X2, X3, Xph, Y (n = 32)	

** *Hyaloraphidium* was included in the Chlorophytes phytoplankton group in this study even if it is now considered as a fungi taxon.
